# Effects of core strength training using stable versus unstable surfaces on physical fitness in adolescents: a randomized controlled trial

**DOI:** 10.1186/2052-1847-6-40

**Published:** 2014-12-15

**Authors:** Urs Granacher, Jörg Schellbach, Katja Klein, Olaf Prieske, Jean-Pierre Baeyens, Thomas Muehlbauer

**Affiliations:** Division of Training and Movement Sciences, Research Focus Cognition Sciences, University of Potsdam, Am Neuen Palais 10, Building 12, 14469 Potsdam, Germany; University College Physiotherapy Thim van der Laan, Landquart, Switzerland; Faculty of Physical Education and Physical Therapy, Free University of Brussels, Brussels, Belgium

**Keywords:** Resistance training, Trunk muscle strength, Physical fitness

## Abstract

**Background:**

It has been demonstrated that core strength training is an effective means to enhance trunk muscle strength (TMS) and proxies of physical fitness in youth. Of note, cross-sectional studies revealed that the inclusion of unstable elements in core strengthening exercises produced increases in trunk muscle activity and thus provide potential extra training stimuli for performance enhancement. Thus, utilizing unstable surfaces during core strength training may even produce larger performance gains. However, the effects of core strength training using unstable surfaces are unresolved in youth. This randomized controlled study specifically investigated the effects of core strength training performed on stable surfaces (CSTS) compared to unstable surfaces (CSTU) on physical fitness in school-aged children.

**Methods:**

Twenty-seven (14 girls, 13 boys) healthy subjects (mean age: 14 ± 1 years, age range: 13–15 years) were randomly assigned to a CSTS (n = 13) or a CSTU (n = 14) group. Both training programs lasted 6 weeks (2 sessions/week) and included frontal, dorsal, and lateral core exercises. During CSTU, these exercises were conducted on unstable surfaces (e.g., TOGU© DYNAIR CUSSIONS, THERA-BAND© STABILITY TRAINER).

**Results:**

Significant main effects of Time (pre vs. post) were observed for the TMS tests (8-22%, *f* = 0.47-0.76), the jumping sideways test (4-5%, *f* = 1.07), and the Y balance test (2-3%, *f* = 0.46-0.49). Trends towards significance were found for the standing long jump test (1-3%, *f* = 0.39) and the stand-and-reach test (0-2%, *f* = 0.39). We could not detect any significant main effects of Group. Significant Time x Group interactions were detected for the stand-and-reach test in favour of the CSTU group (2%, *f* = 0.54).

**Conclusions:**

Core strength training resulted in significant increases in proxies of physical fitness in adolescents. However, CSTU as compared to CSTS had only limited additional effects (i.e., stand-and-reach test). Consequently, if the goal of training is to enhance physical fitness, then CSTU has limited advantages over CSTS.

**Trial registration:**

ClinicalTrials.gov Identifier: NCT02290457 Registered 13 November 2014.

## Background

Core muscle strength is an important prerequisite for several sport (e.g., track and field, climbing, soccer), and everyday activities (e.g., sitting, standing, walking in an upright position). Anatomically, the core can be described as a muscular box with the abdominals in the front, paraspinals and glutes in the back, the diaphragm as the roof, and the pelvic floor and hip girdle musculature as the bottom [1]. Functionally, the core can be thought of as the kinetic link that facilitates the transfer of torques and angular momentum between the lower and upper extremities that is of vital importance for sport-specific and everyday activities in different age groups [2]. In fact, data from a cross-sectional study indicate significant relationships between variables of core muscle strength, sprint, throw, and jump performance in young healthy individuals [3, 4]. With reference to these findings, it seems plausible to argue that core strength training may have the potential to improve core muscle strength as well as health-related (i.e., strength, flexibility) and skill-related (i.e., balance, coordination, speed) components of physical fitness in youth. To the best of our knowledge, there is only one study available that investigated the impact of a 6-week core conditioning program in healthy untrained school-aged children [5]. As a result, the authors found significant performance enhancements in different trunk muscle endurance tests.

Performance of several everyday and sports-related activities occurs on relatively unstable surfaces (e.g., walking on cobblestone pavement, jumping on uneven natural turf, landing on sand during beach-volleyball, kicking a ball while being impeded by an opponent). Thus, according to the concept of training specificity, training must attempt to closely address the demands of these activities. In this regard, Behm and Colado-Sanchez [6] propagated strength training using unstable surfaces and/or devices for performance enhancement and musculoskeletal health in youth and old adults. In a recent study, Granacher et al. [7] conducted a 9 week progressive core strength training on unstable surfaces in community-dwelling old adults (age: 63–80 years). Compared to a passive control group, the intervention group significantly improved measures of trunk muscle strength (TMS), spinal mobility, functional mobility, and dynamic balance. It was concluded that core strength training conducted on unstable surfaces is a feasible and effective exercise program for attenuating age-related performance decrements in old adults. However, in this study core strength training has been conducted on unstable surfaces only. Thus, this study was not able to elucidate the potential additive effect of core strength training using unstable surfaces as compared to core strength training on stable surfaces. Of note, the application of unstable surfaces during youth strength training might be particularly beneficial because balance and coordination are not yet fully developed in school-aged children [8]. Furthermore, the inclusion of unstable elements in strength training exercises leads to substantial force decrements while at the same time overall muscle activity appears to remain unchanged [9]. However, there is evidence in the literature [10] that reduced loads combined with high repetitions still represent a sufficient training stimulus in youth which is why strength training performed on unstable surfaces seems to be well-suited for the promotion of health-related and skill-related components of physical fitness in youth. However, as of now there is no study available that compared the effects of core strength training performed on stable surfaces (CSTS) with core strength training performed on unstable surfaces (CSTU) in youth. In an attempt to fill this void in the literature, we specifically studied the effects of CSTU versus CSTS on health-related and skill-related components of physical fitness in youth.

Based on study findings mentioned above [3–6, 10–12], we hypothesized that participants performing CSTU as compared to CSTS will show larger improvements in physical fitness tests (i.e., strength, speed, flexibility, coordination, balance) following core strength training. Of note, training induced gains in strength, speed, flexibility, coordination, and balance are of vital importance for sports performance, everyday activities, and injury prevention.

## Methods

To test our hypothesis, adaptations following CSTS as compared to CSTU were assessed using a parallel group randomized controlled study design that included pre- and post-testings and core strength training in between. The training period lasted 6 weeks to induce training-related changes in measures of strength, speed, flexibility, coordination, and balance. These health-related (i.e., strength, flexibility) and skill-related (i.e., balance, coordination, speed) components of physical fitness [13] were assessed using physical fitness tests (i.e., Bourban TMS test, standing long jump test, 20-m sprint test, stand-and-reach test, jumping sideways test, Emery balance test, Y balance test).

### Participants

**Figure 1:**
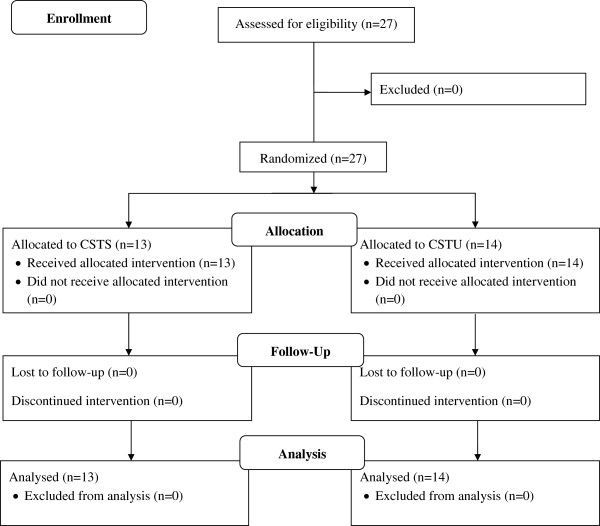
**Flow chart of the progress through the phases of the study according to the CONSORT statements.**

**Figure 2:**
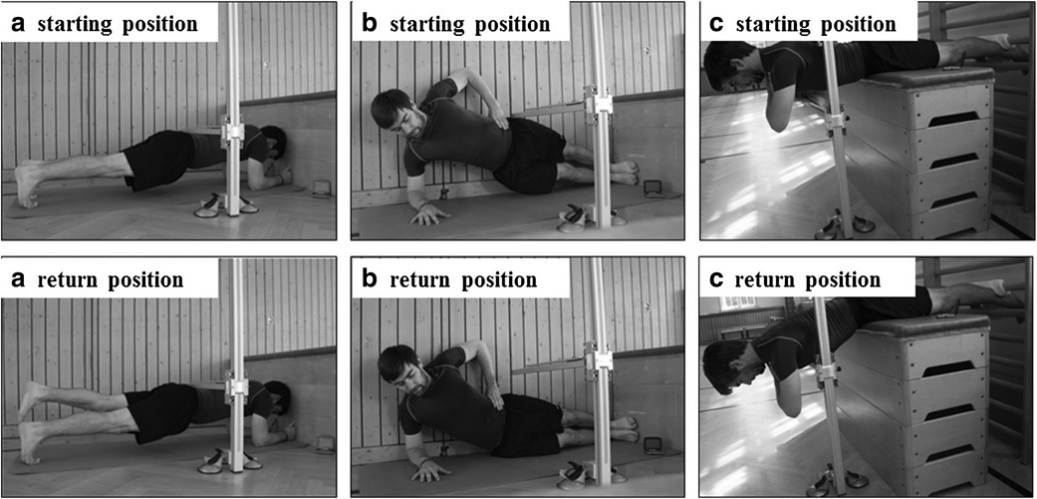
**Schematic description of the Bourban trunk muscle strength test (a: the ventral trunk muscle chain test, b: the lateral trunk muscle chain test, c: the dorsal trunk muscle chain test).**

Twenty-seven healthy boys and girls participated in this study after the experimental procedures were explained. Figure 1 shows a flow chart of the study design. An a priori power analysis [14] with an assumed Type I error of 0.05 and a Type II error rate of 0.20 (80% statistical power) was calculated for measures of trunk muscle strength [12] and revealed that 13 participants per group would be sufficient to observe medium “Time x Group” interaction effects. Study participants were recruited from local sports clubs between May and June 2014. All participants can be classified as physically active according to the Freiburg questionnaire of everyday and sports-related activities [15]. All subjects were advised not to decrease or increase their daily sport activities over the course of the study. Characteristics of the study population are described in Table 1. All participants were eligible for inclusion in this study because they had no history of musculoskeletal, neurological or orthopaedic disorders that might have affected their ability to perform physical fitness tests and core strength training. Further, none had previously participated in systematic strength or balance training. Subjects were randomly assigned to one of 2 intervention (i.e., CSTS or CSTU) using the method of randomly permuted blocks using Research Randomizer, a program published on a publicly accessible official website (http://www.randomizer.org). Two independent experimenter (JS, KK) generated the random allocation sequence, enrolled participants, and assigned participants to the intervention groups. Group 1 conducted a CSTS program under stable conditions whereas group 2 performed CSTU. Parents’ and participants’ informed consents were obtained before the start of the study. Ethical permission was given by the ethics committee of the University of Potsdam (submission No. 26/2014) and all experiments were conducted according to the latest version of the declaration of Helsinki. Written informed consent was obtained from the participant for publication of Figure 2a-c. A copy of the written consent is available for review by the Editor of this journal.

**Table 1:** **Characteristics of the study participants**

Characteristic	CSTS (***n*** = 13)		CSTU (***n*** = 14)		
	***M***	***SD***	***M***	***SD***	***p***-value
Age (years)	13.7	0.6	13.8	0.9	.758
Body height (cm)	168.6	9.7	169.6	9.3	.796
Body mass (kg)	53.1	9.6	51.4	7.3	.602
Body mass index (kg/m^2^)	18.6	2.4	17.8	1.5	.309
Sex (f/m)	7/6	7/6	7/7	7/7	
Physical activity (h/week)	7.1	2.9	6.8	2.4	.721
Leg length left (cm)	79.9	6.5	78.7	4.6	.567
Leg length right (cm)	79.5	6.3	78.3	4.4	.575

*Note*. f = female; m = male; M = mean; SD = standard deviation; CSTS = core strength training on stable surfaces; CSTU = core strength training on unstable surfaces.

### Procedures

#### Core strength training

Both core strength training programs were supervised and conducted by 2 experienced physiotherapists. Thus, the participant-to-supervisor ratio was kept small for both intervention groups with 2 supervisors to 13 participants in the CSTS group and 2 supervisors to 14 participants in the CSTU group. The two programs were organized as circuit training with each instructor supervising 6–7 participants. Both training programs lasted 6 weeks and comprised 2 training sessions per week with a total of 12 training sessions for each intervention group. Each training session lasted 30 min, starting with a brief, standardized warm-up program mainly consisting of low-intensity core strength exercises to prepare the neuromuscular system for the training loads and ending with a cool-down program (i.e., dynamic stretching). During the main part of training, both groups mainly conducted the “big 3” exercises as described by McGill [16]. These include the curl-up, side bridge, and quadruped position. In other words, every single training session consisted of frontal, dorsal, and lateral core exercises. The only difference between the 2 intervention groups was that the CSTU protocol comprised core exercises that were conducted on unstable surfaces (i.e., TOGU© DYNAIR PRO, SENSO, TOGU© REDONDO BALLS, TOGU© POWERBALLS, THERA-BAND© STABILITY TRAINER, THERA-BAND© EXERCISE BALL), whereas the CSTS program contained the same exercises on stable surface only. Table 2 illustrates a detailed description of the core exercises. The CSTU protocol has recently been published [7].

**Table 2:** **Description of the two core strength training programs**

The “big 3” core exercises	Core strength training on stable surfaces	Core strength training on unstable surfaces
*Cross curl-ups*	*Basic exercise position and execution of exercise:* subjects were lying in supine position, hands folded in the neck, elbows pointed to the sides, knees in a flexed position, feet rested on a fitness mat; subjects curled-up until the scapulae left the fitness mat, subjects rotated to the left and right at a moderate movement velocity; *progression during training:* by increasing contraction time (see text), by lifting the feet up in the air at a 90° knee angle	*Basic exercise position and execution of exercise:* same as during the stable condition; additionally, subjects were sitting on a Togu© Dynair cussion and each foot rested on a basketball; *progression during training:* by increasing contraction time (see text), by alternately extending the arms from behind the neck
*Side bridge (both sides)*	*Basic exercise position and execution of exercise:* subjects were lying in a side position with knees flexed, the supporting shoulder superior to the respective elbow, the uninvolved arm held akimbo, and the supporting forearm flat on the fitness mat; subjects raised their hips until a straight line was reached from the knees up to the shoulders, subjects continuously raised and lowered their hips at a moderate movement velocity; *progression during training:* by increasing the number of repetitions (see text), by extending the legs so that a straight line was reached over the whole body, and by lifting the upper leg up in the air	*Basic exercise position and execution of exercise:* same as during the stable condition; additionally, a Togu© Redondo ball with a diameter of 22 cm was placed underneath the subjects’ knees; *progression during training:* by increasing the number of repetitions (see text), by placing a Togu© Redondo ball underneath the feet; by placing a basketball underneath the supporting forearm
*Quadrupedal stance (“birddog exercise”)*	*Basic exercise position and execution of exercise:* subjects started in a quadrupedal stance with both hands and knees flat on the surface; subjects lifted a leg and the contralateral arm in horizontal position; subjects alternately lifted and lowered their leg and contralateral arm at a moderate movement velocity; *progression during training:* by increasing the number of repetitions (see text)	*Basic exercise position and execution of exercise:* same as during the stable condition; additionally, a basketball was placed underneath the supporting hand; *progression during training:* by increasing the number of repetitions (see text), by placing a Togu© Redondo ball with a diameter of 22 cm underneath the supporting knee; by additionally lifting the foot of the supporting leg off the floor

In general, participants always exercised in pairs so that one subject trained and the other one provided support (i.e., motivation, spotting). Training intensity was progressively and individually increased over the 6-week training program by modulating lever lengths, movement velocity (isometric, dynamic), range of motion (i.e., CSTS and CSTU) and the level of instability (i.e., CSTU). During training weeks 1–2, participants of the CSTS and the CSTU performed the “big 3” exercises with 3 sets per exercise and 40 s contraction time (isometric condition) or 20 repetitions (dynamic condition). During training weeks 3–4, contraction times and repetitions were increased to 45 s or 23 repetitions. During training weeks 5–6, contraction times and repetitions were increased to 50 s or 25 repetitions. The rest between sets was similar to the respective contraction time (e.g., 40 s during weeks 1–2). An additional 2–3 min rest was provided between exercises.

### Testing

All tests were performed in gyms using standardized test protocols. Prior to pre- and post-tests, all participants underwent a standardized 5-minutes warm-up which consisted of bipedal and monopedal balance, submaximal plyometric, and skipping exercises. Thereafter, physical fitness tests (i.e., secondary outcome measures: Emery balance test, Y balance test, stand-and-reach test, 20-m sprint test, jumping sideways test, standing long jump test; primary outcome measures: Bourban TMS test) were assessed. This sequence of measurements was applied to keep the effects of neuromuscular fatigue minimal during pre- and post-testing.

### Bourban TMS test

The Bourban TMS test assesses core strength endurance of ventral, lateral, and dorsal trunk muscle chains. Tests were applied in randomized order with a 10 min rest between the tests. During *the ventral trunk muscle chain test*, subjects were in prone bridge position on their elbows and toes (Figure 2a). Legs were extended, elbows shoulder-widths apart, and forearms lay flat on a fitness mat. In this test position, the glenohumeral joint, the greater trochanter, and the lateral malleolus were located on a straight line. An adjustable alignment device was constructed that consisted of a stable vertical pole with two vertically adjustable horizontal rods [17]. While in the bridged position, the lower horizontal reference rod of the alignment device was moved into contact with the participant’s lower back at the level of the iliac crests and was then fixed at this position. After visual inspection of the subjects’ starting position, they were asked to lift their feet alternately for 2–5 cm according to the beat of a metronome (1 s per foot). Before the test started, subjects were instructed to remain in contact with the horizontal reference rod for as long as possible. Warnings were given when subjects lost touch to the horizontal rod. The test was terminated when participants failed to remain in contact with the reference rod for the third time. Contact time until test termination was taken as dependent variable and used for further analysis. According to recommendations regarding absolute reliability [18], the ventral test can be classified as reliable with a coefficient of variation of 14.1% [19]. During *the lateral trunk muscle chain test*, subjects were in a side bridge position with legs extended, the upper foot placed on top of the lower foot, and the supporting shoulder superior to the respective elbow (Figure 2b). The supporting forearm was placed flat on the fitness mat and the uninvolved arm was held akimbo. The test was performed in randomized order for the right and left side. Subjects raised their hips until a straight line was reached from the ankles up to the shoulders. While in the side bridged position, the lower horizontal reference rod of the alignment device was fixed at the height of the superior iliac crest. After visual inspection of the subjects’ starting position, participants continuously raised and lowered their hips to the beat of a metronome (2 s per lowering and lifting cycle). They were not allowed to unload their body mass on the fitness mat during the lowering phase. Warnings were given when subjects lost touch to the horizontal rod or when they unloaded their body mass on the fitness mat. The test was terminated when participants received the third warning. Time until test termination was taken as dependent variable and used for further analysis. According to recommendations regarding absolute reliability [18], the lateral test can be classified as reliable with a coefficient of variation (CoV) of 14.6% [19]. During *the dorsal trunk muscle chain test*, subjects lay prone on a wooden box while maintaining an unsupported trunk (from the upper border of the iliac crest) (Figure 2c). Participants held their arms across the chest, hands rested on the shoulders, legs were extended, and the feet were firmly fixed in wall bars. The horizontal position (0°) was controlled using a mechanical goniometer. While in this position, the upper horizontal reference rod of the alignment device was fixed at the level of a thoracic spinal process. Thereafter, the subject lowered the trunk by 30° which was again controlled by a mechanical goniometer. While in this position, the lower horizontal reference rod of the alignment device was fixed at the level of the sternal angle. After visual inspection of subjects’ starting position, participants continuously raised and lowered their trunk to the beat of a metronome (2 s per lowering and lifting cycle). The test was terminated when participants failed to reach the upper horizontal rod for the third time. Time until test termination was taken as dependent variable and used for further analysis. According to recommendations regarding absolute reliability [18], the dorsal test can be classified as reliable with a CoV of 11.7% [19].

### Standing long jump test

The standing long jump test has been considered a general index of muscular fitness in youth [20]. Before the test started, subjects were instructed to stand with both feet right behind a starting line and to jump as far as possible. Subjects were allowed to use arm swing during the test. Three trials were performed with a 2 min rest between trials. The best trial in terms of maximal distance from the starting line to the landing point at heel contact was used for statistical analysis. Measurements were taken to the nearest cm using a tape measure. The standing long jump test has been reported to be reliable with a CoV of 2.4% [21].

### 20-m sprint test

Maximum effort sprints were assessed from a stationary start. Subjects were instructed to stand with one foot right behind the starting line and to accelerate at maximum effort to the finish line. The best out of 3 trials (i.e., minimal sprint time) with a 2 min rest between trials was used for further data analysis. Time was taken with a stop watch to the nearest 1/100 s. Excellent test-retest reliability has been reported for the hand stopped 20-m sprint test with an intraclass correlation coefficient (ICC) of 0.90 [22].

### Stand-and-reach test

Spinal and pelvic flexibility was tested using the stand-and-reach test. Subjects were instructed to begin the test in a standing position on an elevated platform with feet together. They were asked to bend over using their maximal range of motion. During the test, knees, arms, and fingers were fully extended. A tape measure was attached to the platform with 100 cm indicating the top level of the platform. Values >100 cm indicate that the person was able to reach beyond the toes (i.e., good flexibility). Values <100 cm indicate that the person was not able to reach the toes (i.e., limited flexibility). The maximum reach distance was taken as dependent variable. Two trials with a 1 min rest between trials were performed. Excellent test-retest reliability has been reported for the stand-and-reach test with an ICC of 0.94 [22].

### Jumping sideways test

The jumping sideways test evaluates motor coordination under time pressure [23]. Subjects were instructed to jump as many times as possible over a period of 15 s with both legs together back and forth across a strip of wood that was attached to a mat (50 × 100 cm). The number of jumps completed without touching the strip and without stepping off the mat was taken as dependent variable. Two trials with a 2 min rest between trials were performed and the mean of the 2 trials was taken for further analysis. Excellent test-retest reliability has been reported for the jumping sideways test with an ICC of 0.89 [22].

### Emery balance test

The Emery balance test was conducted barefooted in single leg stance on an Airex balance pad. Eyes were closed and both legs were tested [24]. For experimental testing, participants were asked to stand as stable as possible with the knee of the weight-bearing limb flexed at 30°. The non-weight-bearing limb was flexed 45° at the knee and hands were placed on hips. Using a stopwatch, time was stopped upon loss of balance to the nearest 1/100 of a second and used as dependent variable. Loss of balance included removal of one hand from the hip, touching the balance pad or floor with the non-weight-bearing foot, movement of the weight-bearing foot from its original position on the balance pad, movement of the balance pad from its original position during the balance test, or when eyes were opened [24]. Three trials were completed on each leg with 15 s rest between trials. The best trial in terms of maximum standing time was taken for further analysis. Adequate test-retest reliability has been reported for the Emery balance test with an ICC of 0.59 [24].

### Y balance test

The lower quarter Y balance test is a dynamic test that requires subjects to maintain single leg stance while reaching as far as possible with the contralateral leg in 3 different movement directions (i.e., anterior, posteromedial, posterolateral) [25]. For this purpose, a grid consisting of 3 lines was constructed on a gym floor using a mechanical goniometer and adhesive tape measure. The 2 posterior lines extended from the centre of the grid and were positioned 135° from the anterior line with 45° between the two posterior lines. Each line was marked in 5 mm increments for measurement purposes. Before the test started, participants’ length of the right and left leg were assessed in supine lying position by measuring the distance from the anterior superior iliac spine to the most distal aspect of the medial malleolus. Further, subjects practiced 6 trials per reach direction on each foot to get familiarized with the testing procedures. All trials were conducted barefooted. According to Plisky et al. [25], subjects always started with the right foot placed at the centre of the grid and the left leg reaching in anterior direction as far as possible, lightly touching the farthest point possible on the line with the most distal part of the reach foot. Participants then returned to a bilateral stable stance position. After 3 reaches, the left foot was placed at the centre of the grid and the right leg maximally reached in anterior direction. Thereafter, the same test procedure was conducted for the posteromedial and the posterolateral reach. Between reaches, a rest of 15 s was allowed. The examiner manually measured the distance from the centre of the grid to the touch point and the results were documented after each reach. Trials were discarded and repeated if the participant (1) did not touch the line with the reach foot while maintaining weight bearing on the stance leg, (2) lifted the stance foot from the centre grid, (3) lost balance at any point during the trial, (4) did not maintain start and return positions for one full second, or (5) touched down the reach foot to gain considerable support. For further data analyses, the mean of 3 successful reaches was used for each leg in each of the 3 directions. According to Filipa et al. [26], a composite score (CS) was calculated and taken as dependent variable using the following formula: CS = [(maximum anterior reach distance + maximum posteromedial reach distance + maximum posterolateral reach distance)/(leg length × 3)] × 100. Excellent test-retest reliability has been reported for the Y balance test in all 3 movement directions with ICC values ranging between 0.89 and 0.93 [25].

### Statistical analyses

Data are presented as group mean values and standard deviations. Given that we could not detect statistically significant differences between males and females (*p* > 0.05), data were pooled for males and females. A multivariate analysis of variance (MANOVA) was used to detect differences between study groups in all baseline variables. The effects of core strength training on variables of physical fitness were analysed in separate 2 (Group: CST, CSTU) × 2 (Time: pre, post) ANOVA with repeated measures on “Time”. Bonferroni corrections were not necessary because our study design (2 × 2) did not demand multiple testing. When “Time x Group” interactions reached the level of significance, group-specific post hoc tests (i.e., paired *t*-tests) were conducted to identify the comparisons that were statistically significant. Additionally, the classification of effect sizes (*f*) was determined by calculating partial *η*^*2*^_p._ According to Cohen [27], 0.00 ≤ *f* ≤ 0.24 indicate small effects, 0.25 ≤ *f* ≤ 0.39 indicate medium effects, and *f* ≥ 0.4 indicate large effects. The significance level was set at *p* < 0.05. Tendencies towards significance were denoted as 0.051 ≤ *p* < 0.1. All analyses were performed using Statistical Package for Social Sciences (SPSS) version 22.0.

## Results

All subjects received treatment conditions as allocated. Twenty-seven participants completed the training program and none reported any training-related injury. Mean attendance rates at training sessions amounted to 81% for the CSTS group and 83% for the CSTU group. Table 3 describes pre and post intervention results for all outcome variables. Overall, there were no statistically significant differences in baseline values between the 2 intervention groups (*p* > 0.05).

**Table 3:** **Effects of the two core strength training programs on measures of physical fitness**

	CSTS (***n*** = 13)					CSTU (***n*** = 14)					***p***-value (effect size ***f***)		
Variables	Pre		Post		Δ (%)	Pre		Post		Δ (%)	Main effect: Time	Main effect: Group	Interaction: Time × Group
	***M***	***SD***	***M***	***SD***		***M***	***SD***	***M***	***SD***				
*Strength*													
Ventral TMS test (s)	65.5	37.0	74.7	39.3	14.0	67.9	34.1	83.1	28.7	22.4	.001 (.76)	.685 (.08)	.353 (.19)
Dorsal TMS test (s)	152.2	98.0	214.8	32.8	41.1	129.9	55.0	173.3	20.4	33.4	.100 (.34)	.572 (.11)	.758 (.06)
Lateral right side TMS test (s)	46.9	18.9	51.1	18.3	9.0	46.7	12.1	50.4	14.7	7.9	.103 (.34)	.937 (.00)	.913 (.00)
Lateral left side TMS test (s)	46.5	20.8	51.4	18.7	10.5	47.6	10.3	51.4	10.6	8.0	.028 (.47)	.921 (.00)	.746 (.06)
Standing long jump (cm)	187.6	47.4	189.6	39.0	1.1	201.1	20.0	207.1	18.8	3.0	.061 (.39)	.230 (.25)	.336 (.20)
*Speed*													
20-m sprint (s)	3.9	0.4	3.8	0.4	-2.6	3.7	0.2	3.7	0.2	0	.311 (.21)	.434 (.16)	.358 (.19)
*Flexibility*													
Stand-and-reach test (cm)	102.3	9.8	102.0	9.8	-0.3	99.6	8.7	101.5	8.2	1.9	.062 (.39)	.647 (.09)	.012 (.54)
*Motor coordination under time pressure*													
Jumping sideways (# of jumps)	45.8	7.9	49.5	5.0	8.1	48.9	4.0	53.8	4.4	10.0	<.001 (1.07)	.068 (.38)	.477 (.14)
*Balance*													
Emery test on right leg (s)	9.0	4.8	7.9	3.9	-12.2	10.4	7.1	9.2	5.2	-11.5	.378 (.18)	.436 (.16)	.979 (.00)
Emery test on left leg (s)	10.3	7.2	10.5	7.9	1.9	8.9	4.6	10.7	6.2	20,2	.418 (.16)	.772 (.05)	.527 (.13)
Y balance test CS on right stance leg (%)	119.9	12.0	123.3	10.9	2.8	122.2	10.3	124.5	8.7	1.9	.032 (.46)	.656 (.09)	.662 (.09)
Y balance test CS on left stance leg (%)	120.2	12.2	123.4	11.7	2.7	122.5	10.3	124.5	8.2	1.6	.022 (.49)	.680 (.08)	.554 (.12)

*Note*. M = mean; SD = standard deviation; CS = composite score; CSTS = core strength training on stable surfaces; CSTU = core strength training on unstable surfaces; TMS = trunk muscle strength; # = number; the CS for the Y balance test was calculated according to the following formula: CS = [(maximum anterior reach distance + maximum posterior medial reach distance + maximum posterior lateral reach distance)/(leg length × 3)] × 100 [26].

### Bourban TMS test

**Figure 3:**
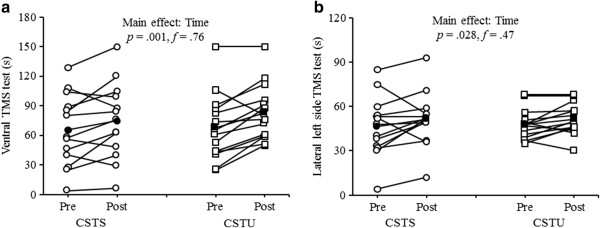
**Individual and mean pre- and post-testing data for a) ventral trunk muscle strength (TMS) test and b) lateral left side TMS test by intervention group (CSTS, core strength training program using stable surfaces; CSTU, core strength training using unstable surfaces).** Unfilled circles indicate individual data of the CSTS-group and filled circles indicate mean data of the CSTS-group. Unfilled squares indicate individual data of the CSTU-group and filled squares indicate mean data of the CSTU-group.

The statistical analysis indicated significant main effects of “Time” for the ventral TMS test (*F*_1, 25_ = 14.51, *p* < 0.001, *f* = 0.76) and the lateral left side TMS test (*F*_1, 25_ = 5.48, *p* < 0.05, *f* = 0.47) (Figure 3a, b). Further, trends towards significant main effects of “Time” were observed for the dorsal TMS test (*F*_1, 25_ = 2.91, *p* = 0.10, *f* = 0.34) and the lateral right side TMS test (*F*_1, 25_ = 2.86, *p* = 0.10, *f* = 0.34). However, we could not detect a significant main effect of “Group” nor a “Time × Group” interaction (Table 3).

### Standing long jump test

A tendency towards a significant main effect of “Time” was found for the standing long jump test (*F*_1, 25_ = 3.85, *p* = 0.061, *f* = 0.39). Yet, no significant main effect of “Group” nor a “Time × Group” interaction were found (Table 3).

### 20-m sprint test

Our statistical calculations revealed no significant main effects of “Time” and “Group” and no significant “Time x Group” interaction for the 20-m sprint test (Table 3).

### Stand-and-reach test

**Figure 4:**
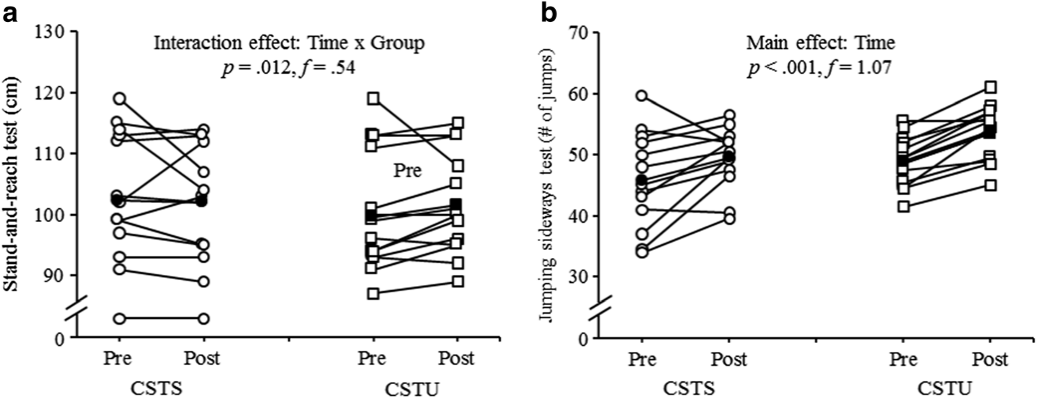
**Individual and mean pre- and post-testing data for a) stand-and-reach test and b) jumping sideways test by intervention group (CSTS, core strength training program using stable surfaces; CSTU, core strength training using unstable surfaces).** Unfilled circles indicate individual data of the CSTS-group and filled circles indicate mean data of the CSTS-group. Unfilled squares indicate individual data of the CSTU-group and filled squares indicate mean data of the CSTU-group.

A trend towards a significant main effect of “Time” (*F*_1, 25_ = 3.83, *p* = 0.062, *f* = 0.39) but not of “Group” was detected for the stand-and-reach test (Figure 4a, Table 3). In addition, a significant “Time × Group” interaction was found (*F*_1, 25_ = 7.28, *p* = 0.012, *f* = 0.54). Post-hoc analysis revealed a significant increase in maximal reach distance from pre- to post-test in the CSTU group (Δ 2%, *p* < 0.01).

### Jumping sideways test

A significant main effect of “Time” (*F*_1, 25_ = 28.75, *p* < 0.001, *f* = 1.07) and a trend towards a significant main effect of “Group” (*F*_1, 25_ = 3.65, *p* = 0.068, *f* = 0.38) was found for the jumping sideways test. We could not detect a significant “Time × Group” interaction (Figure 4b, Table 3).

### Emery balance test

Our statistical analyses revealed no significant main effects of “Time” and “Group” and no significant “Time × Group” interactions for the Emery balance performed with the right and the left leg (Table 3).

### Y balance test

**Figure 5:**
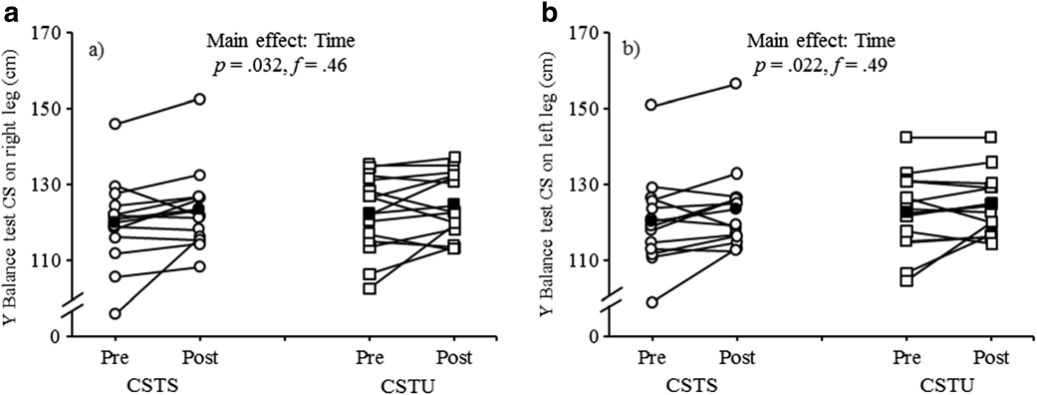
**Individual and mean pre- and post-testing data for a) Y balance test composite score (CS) while standing on the right leg and b) Y balance test CS while standing on the left leg by intervention group (CSTS, core strength training program using stable surfaces; CSTU, core strength training using unstable surfaces).** Unfilled circles indicate individual data of the CSTS-group and filled circles indicate mean data of the CSTS-group. Unfilled squares indicate individual data of the CSTU-group and filled squares indicate mean data of the CSTU-group.

Significant main effects of “Time” were investigated regarding the CS when performing the Y balance test on the right (*F*_1, 25_ = 5.19, *p* < 0.05, *f* = 0.46) and the left leg (*F*_1, 25_ = 5.98, *p* < 0.05, *f* = 0.49) (Figure 5a, b). Yet, no significant main effect of “Group” nor “Time × Group” interactions were investigated (Table 3).

## Discussion

To the authors’ knowledge, this is the first study that investigated the effects of CSTS compared to CSTU on health and skill-related components of physical fitness in healthy youth. The main findings of this study were that *(1)* performance in physical fitness tests (i.e., Bourban TMS test, standing long jump test, stand-and-reach test, jumping sideways test, Y balance test) significantly improved in both intervention groups over the 6-week training period; *(2)* CSTU as compared to CSTS has only limited additional effects (i.e., stand-and-reach test) on physical fitness.

The present results are in accordance with the literature regarding the effects of core strength training on TMS and physical fitness in youth. Following 6 weeks of core strength training (e.g., low plank obliques, push-up jacks) conducted during physical education classes (1 session/week), Allen et al. [5] found significant performance enhancements (*f* = 0.27-0.69) in 5 different trunk muscle endurance tests (i.e., Parallel Roman Chair Dynamic Back Extension, Prone Plank, Lateral Plank, Dynamic Curl-Up, Static Curl-up) in healthy untrained children with a mean age of 11 years. In a randomized controlled trial, Hoshikawa et al. [12] investigated the effects of a combined core strength training (e.g., prone and side bridging on elbows) and soccer training (e.g., technical drills, interval runs) as compared to soccer training only (e.g., technical drills, interval runs) in male outfield soccer players aged 12–13 years. Both intervention groups exercised for 6 months. The combined training group conducted 4 core strength training and 5 soccer training sessions per week, whereas the soccer training group performed 5 soccer training sessions per week only. Before and after training, subjects were tested for their hip flexors/extensors strength, cross-sectional area of trunk muscles, and athletic performance. With respect to hip strength and physical fitness measures, both intervention groups showed significant but similar performance enhancements in peak torque of the hip flexors (combined training group: *f* = 0.45, isolated training group: *f* = 0.74) and in 15-m sprint test (combined training group: *f* = .56, isolated training group: *f* = 0.40) following training. However, the relative change in peak hip extensor torque was significantly higher in the combined (*f* = 1.26) as compared to the isolated (*f* = 0.68) training group. Furthermore, significant gains in squat (combined training group: *f* = 0.33, soccer training group: *f* = 0.06) and countermovement jump heights (combined training group: *f* = 0.62, soccer training group: *f* = 0.12) were observed in the combined training group only.

Our findings extend the existing results in as much as we additionally observed improvements in measures of flexibility, coordination, and balance following core strength training in youths. With reference to the literature [5, 12] and our own findings, core strength training appears to be a well-suited conditioning program for the promotion of health-related and skill-related physical fitness in youth. The positive effects of core strength training on physical performance of the lower extremities can most likely be explained by the specific role of the trunk as a linkage between upper and lower extremities. Particularly during every-day or sports-related rotational torso movements, trunk muscles generate torque along a diagonal proximal to distal path to enhance extremity force production. Konin and colleagues [28] referred to this as the so-called serape (i.e., “shawl-like”) effect. Scientific evidence was provided by Kibler [29] who was able to show that 51% of total kinetic energy and 54% of total force are developed in the hip and trunk muscles during the tennis serve of professional athletes. According to Young et al. [30], muscles belonging to the global system (e.g., erector spinae, rectus abdominis, internal/external obliques, latissimus dorsi) primarily generate torque in a serape-like manner during rotational movements (e.g., throwing). Moreover, the trunk acts as a kinetic link that facilitates the transfer of torques and angular momenta between upper and lower extremities during the execution of whole body movements as part of sports and occupational skills, fitness activities, and activities of daily living [31]. There is evidence for this hypothesis which indicates that during normal human movement, trunk muscle activations (e.g., musculus transversus abdominis) are organized well ahead (110 ms) in anticipation of movement or perturbation to balance in healthy young adults [32]. Hodges and Richardson [32] argued that this anticipatory muscle activation helps stiffening the spine to provide a foundation for functional movements. Thus, muscles belonging to the local system (e.g., lumbar multifidus, transversus abdominis) appear to primarily provide proximal stability of the trunk for distal mobility of the limbs. Of note, our core strength training protocols comprising multiple sets with many repetitions or long contraction times may have specifically induced adaptive processes in muscles of the local system (deep muscles) since those muscles are characterized by a relatively high proportion of type I (slow-twitch) fibers [31]. Interestingly, performance during physical fitness tests significantly improved although postural positions during training and testing conditions were different (i.e., horizontal lying during training vs. upright standing during testing). Despite this difference, transfer effects were notified from core strength exercises performed in vertical directions while lying in horizontal positions to proxies of physical fitness predominately performed in vertical position. Future studies have to elucidate whether core strength training programs conducted in an upright standing position (e.g., Romanian deadlift) may be even more effective in enhancing components of physical fitness in adolescents.

By integrating unstable surfaces in our CSTU exercise protocol, we specifically aimed at activating the deep muscles that are responsible for trunk stability. Nevertheless, our findings indicate that CSTU as compared to CSTS has only limited additional effects (i.e., stand-and-reach test) on physical fitness. In this regard, Willardson et al. [33] compared trunk muscle activity (i.e., rectus abdominis, external/internal oblique, transversus abdominis, erector spinae) during resistance exercises (i.e., back squat, dead lift, overhead press, curl lifts) performed on stable ground versus the BOSU Balance Trainer in trained young men. The main finding of this study was that no significant differences were found in activity across all examined muscles and lifts when performing the resistance exercises on the BOSU Balance Trainer as compared to stable ground. The authors concluded that the tested resistance exercises can be performed on stable ground without losing the potential trunk muscle training benefits. Our findings of limited additional effects of CSTU as compared to CSTS are in line with the results of this cross-sectional study. To the authors’ knowledge, there is no other study available in the literature that compared the effects of CSTS versus CSTU on measures of physical fitness. Therefore, we will discuss a study that investigated the effects of lower extremity strength training using stable versus unstable surfaces on athletic performance in healthy, trained individuals [34]. Both intervention groups performed the same exercises (e.g., squats, deadlifts, lunges, single-leg squats) at identical training volumes but on different training surfaces (stable vs. unstable). Following 10 weeks of training, findings were inconsistent in as much as the unstable group showed significantly greater improvements than the stable group in sprint time (stable group: *f* = 1.33, unstable group: *f* = 1.50) and in agility performance (stable group: *f* = 0.97, unstable group: *f* = 1.60). In terms of drop jump power performance, both groups showed similar performance enhancements (stable group: *f* = 0.26, unstable group: *f* = 0.11).

## Conclusions

In summary, the results of this study illustrate that core strength training is a feasible (i.e., high adherence rate of ≥81%) and safe (i.e., no injuries reported) training modality that produces marked increases in health (i.e., strength, flexibility) and skill-related (i.e., balance, coordination, speed) components of physical fitness in healthy male and female youths. Contrary to our hypothesis, CSTU as compared to CSTS has only limited additional effects (i.e., stand-and-reach test) on physical fitness. Consequently, if the goal is to enhance physical fitness, CSTU has no advantage over CSTS.
